# Hypocalcaemia upon arrival (HUA) in trauma patients who did and did not receive prehospital blood products: a systematic review and meta-analysis

**DOI:** 10.1007/s00068-024-02454-6

**Published:** 2024-02-06

**Authors:** Timothy J. Rushton, David H. Tian, Aidan Baron, John R. Hess, Brian Burns

**Affiliations:** 1 School of Medicine Sydney, University of Notre Dame Australia, Sydney, NSW Australia; 2 Department of Anaesthesia and Perioperative Medicine, Westmead Hospital, Sydney, NSW Australia; 3 Department of Surgery, University of Melbourne, Melbourne, VIC Australia; 4 Faculty of Health, Science, Social Care and Education, Kingston University, London, UK; 5 Transfusion Service, Harborview Medical Center, Seattle, WA USA; 6 Department of Laboratory Medicine and Pathology, University of Washington School of Medicine, Seattle, WA USA; 7 Trauma Service, Royal North Shore Hospital, Reserve Rd, St Leonards, Sydney, NSW 2065 Australia; 8 Aeromedical Operations, NSW Ambulance, Sydney, NSW Australia; 9 Sydney Medical School, Sydney University, Sydney, NSW Australia; 10 Faculty of Medicine, Macquarie University, Sydney, NSW Australia

**Keywords:** Hypocalcaemia, Calcium, Prehospital, Trauma, Haemorrhagic shock, Blood transfusion

## Abstract

**Purpose:**

Hypocalcaemia upon arrival (HUA) to hospital is associated with morbidity and mortality in the trauma patient. It has been hypothesised that there is an increased incidence of HUA in patients receiving prehospital transfusion as a result of citrated blood products. This research aimed to determine if there was a difference in arrival ionised calcium (iCa) levels in trauma patients who did and did not receive prehospital transfusion.

**Methods:**

We conducted a systematic review and meta-analysis of patients with an Injury Severity Score (ISS) > / = 15 and an iCa measured on hospital arrival. We then derived mean iCa levels and attempted to compare between-group variables across multiple study cohorts.

**Results:**

Nine studies reported iCa on arrival to ED, with a mean of 1.08 mmol/L (95% CI 1.02–1.13; *I*^2^ = 99%; 2087 patients). Subgroup analysis of patients who did not receive prehospital transfusion had a mean iCa of 1.07 mmol/L (95% CI 1.01–1.14; *I*^2^ = 99%, 1661 patients). Transfused patients in the 3 comparative studies had a slightly lower iCa on arrival compared to those who did not receive transfusion (mean difference − 0.03 mmol/L, 95% CI − 0.04 to − 0.03, *I*^2^ = 0%, *p* = 0.001, 561 patients).

**Conclusion:**

HUA is common amongst trauma patients irrespective of transfusion. Transfused patients had a slightly lower initial iCa than those without transfusion, though the clinical impact of this remains to be clarified. These findings question the paradigm of citrate-induced hypocalcaemia alone in trauma. There is a need for consensus for the definition of hypocalcaemia to provide a basis for future research into the role of calcium supplementation in trauma.

## Introduction

Calcium ions play a vital role in various physiological processes relevant to the shocked trauma patient. It is an essential electrolyte responsible for cardiovascular function through enabling cardiomyocyte contraction upon its release from the sarcoplasmic reticulum [1, 2] and by maintenance of vascular tone [3]. Hypocalcaemia is shown to cause acute cardiovascular compromise through the development of dysrhythmias [4], hypotension, as well as decreased cardiac output and contractility [1, 5, 6]. Calcium is also essential in haemostasis and coagulation. The calcium ion acts as a positively charged bridge between negatively charged phospholipids and vitamin K-dependent clotting factors II, VII, IX, and X [7]. Calcium also acts as a secondary messenger in platelet function and has been significantly associated with platelet activation, aggregation, and viscoelastic clot strength [8]. Hypocalcaemia upon arrival (HUA) in trauma patients is associated with higher overall mortality [9–18] and blood product transfusion rates [10, 14, 18, 19]. As a result, calcium has been identified as part of the lethal diamond of death alongside hypothermia, coagulopathy, and acidosis [20, 21]. However, there is no robust evidence of causation over correlation.

Intravenous administration of citrate causes transient hypocalcaemia, after which ionised calcium (iCa) has been shown to return to pre-transfusion levels from a few minutes to hours [22–24]. Citrate is a common anticoagulant that has been used in transfusion medicine since 1914 [25]. A unit of packed red blood cells contains 3 g of citrate which can be metabolised by a healthy adult in 5 min [26]. It is often observed that patients receiving citrated blood products are hypocalcaemic, especially in cohorts of patients undergoing massive transfusion in hospital. As a result, intravenous calcium supplementation has become routine in these patients based on the knowledge that citrated blood products can induce hypocalcaemia in recipient trauma patients.

HUA has also been found to be common amongst shocked trauma patients independent of prehospital blood product administration [27]. This mechanism should be considered in shocked trauma patients.

A better understanding of the mechanisms causing hypocalcaemia in prehospital trauma patients is important as prehospital transfusion is increasingly becoming the standard of care in the bleeding trauma patient in many advanced trauma systems. An Australian study reported the use of prehospital transfusion increased over two-fold from 2010 to 2018 [28]. Prehospital transfusion is also becoming more complex as services are increasingly implementing balanced transfusion practices [29] and early activation of mass transfusion protocols [30].

This is the first systematic review combining a meta-analysis that compares arrival iCa levels in trauma patients who did and did not receive prehospital blood products. The primary aim was comparison of arrival iCa levels in trauma patients who did and did not receive prehospital blood products. The hypothesis was that hypocalcaemia is common in severe trauma patients regardless of whether they received prehospital blood products or not. Secondary aims were to compare the arrival iCa of patients receiving different blood product types and explore associations with injury severity, injury type, mortality, and in-hospital transfusion requirement.

## Methods

The protocol for this review was registered in the International Prospective Register of Systematic Reviews (PROSPERO CRD42022315189). This study’s methodology is reported in accordance with the Preferred Reporting Items for Systematic Reviews and Meta-Analyses (PRISMA) guidelines [31].

### Search strategy

A literature search of the PUBMED, MEDLINE, EMBASE, CINAHL, International Clinical Trials Registry Platform, and Cochrane Library electronic databases was performed on 31 March 2023 containing the following search terms:

("trauma patient* OR "severe trauma*" OR "major trauma" OR "major trauma patient*" OR "adult trauma patient*" OR "traumatic injur*" OR "Wounds AND Injuries"[mesh:noexp]OR "hemorrhagic shock" OR "haemorrhagic shock" OR "hypovolemic shock" OR "major bleed*" OR Shock[mesh:noexp] OR Shock, Hemorrhagic[mesh:noexp] OR Shock, Traumatic[mesh:noexp) AND (hypocalcemia OR hypocalcaemia OR calcium OR "ionised calcium" OR "ionized calcium" OR Hypocalcemia[mesh:noexp] OR Calcium[mesh:noexp]ae, bl, df).

Manual searches of reference lists were also performed and Google Scholar was searched for citations from non-indexed manuscripts. No further limitations to the search strategy were applied due to the scarcity of prehospital literature.

### Inclusion and exclusion criteria

Study types that were included consisted of randomised and non-randomised controlled trials, cohort, cross-sectional, and retrospective studies. Small case series, case reports, reviews, abstracts, ecological studies, and animal studies were excluded. There was no limitation on publication period and studies in languages other than English were included if accompanied by an English translation.

Inclusion criteria for study participants were as follows: trauma patients with a reported mean or median Injury Severity Score (ISS) > / = 15 who had an iCa measured on arrival to hospital. Exclusion criteria for study participants included patients who were not being treated for severe trauma and those who did not have an iCa measured on arrival to hospital. Studies were grouped into those consisting of patients who received prehospital product, those who received no prehospital product, and comparative studies wherein some patients received prehospital product and some did not.

Two reviewers (TR and AB) independently performed title and abstract screening and a third reviewer (BB) was consulted to resolve any discrepancies. This process was performed with the aid of Rayyan [32], a systematic review software program that utilises artificial intelligence to assist in filtering and sorting references.

### Data item and data extraction

Two authors (TR and AB) performed data collection from the full-text articles with the aid of a standardised form. The following data were extracted:

- General study information—publication year, location, study type, the total number of participants
- Participant characteristics—ISS score and injury mechanism (blunt or penetrating)
- Clinical information—prehospital time, on-scene and ED vital signs, shock index, GCS, mortality, prehospital and in-hospital transfusion requirement, and type
- Laboratory information—lactate, INR, base deficit, pH, and arrival iCa.

Arrival iCa was defined as the initial iCa tested upon arrival to hospital via arterial or venous blood gas. Severe trauma was defined as a reported mean or median ISS > / = 15. Where required, study authors were contacted for clarification or further data provision.

### Quality assessment

Risk of bias assessment was manually performed by 2 independent authors (TR and AB). The ROB-2 [33] and Newcastle–Ottawa Scale (NOS) tools [34] were used for assessing randomised and non-randomised trials respectively. The ROB-2 tool grades studies as being either “high risk” of bias, “low risk” of bias, or having “some concerns.” The NOS grades studies from 0 to 9 stars based on a studies selection, comparability, and outcome. Studies that scored > / = 7 were deemed to be “good” quality, 2–6 “fair” quality, and < 2 “poor” quality.

### Statistical analysis

The primary outcome of interest was iCa level on arrival to hospital. Secondary outcome measures were too heterogenous and incomplete to allow robust statistical analysis with the exception of deriving a mean ISS amongst the cohort of studies included. Analysis for the primary outcome was performed for all patients, as well as the subset of patients who did not receive prehospital blood transfusion. Comparative analysis of prehospital blood transfusion and no transfusion was also performed if studies reported both groups. iCa reported as median or a range were converted to mean and standard deviation (SD) to facilitate meta-analysis using the methods of Luo et al. [35] and Wan et al. [36].

iCa concentration was pooled using the Hartung-Knapp-Sidik-Jonkman random-effects meta-analysis of proportions for both overall aggregation and between-group comparisons [37]. A Bayesian meta-analysis was further performed to explore the robustness of the results of the between-group comparison, noting the likely scarcity of studies. The Bayesian meta-analysis was performed using vague priors (zero for mu and half-normal with scale 0.5 for tau). Results are presented as mean difference (MD) to provide clinically interpretable values. 95% confidence intervals (CIs) and 95% credible intervals (CrI) are also presented. *P* values less than 0.05 were considered to be significant.

Quantitative heterogeneity was assessed using *I*^2^. If *I*^2^ > 50%, suggesting significant heterogeneity, then study-level characteristics (study size, median year of recruitment, continent of practice) were explored with meta-regression to identify contributions to heterogeneity.

All statistical analysis was performed with R (version 4.1.0, R Foundation for Statistical Computing, Vienna, Austria), and packages *meta* (version 6.2–1) and *bayesmeta* (version 3.2).

## Results

### Description of systematic review search

Out of 2435 unique citations 34 full-text articles were reviewed by two authors (TR and BB) resulting in 14 being included in the systematic review [8–11, 14–16, 19, 38–43], three of which were included in the primary meta-analysis [15, 39, 40]. The remaining 20 studies were excluded due to the timing of iCa measurement, absence of iCa measurement, incorrect population, or absence of a full text.

**Fig. 1:**
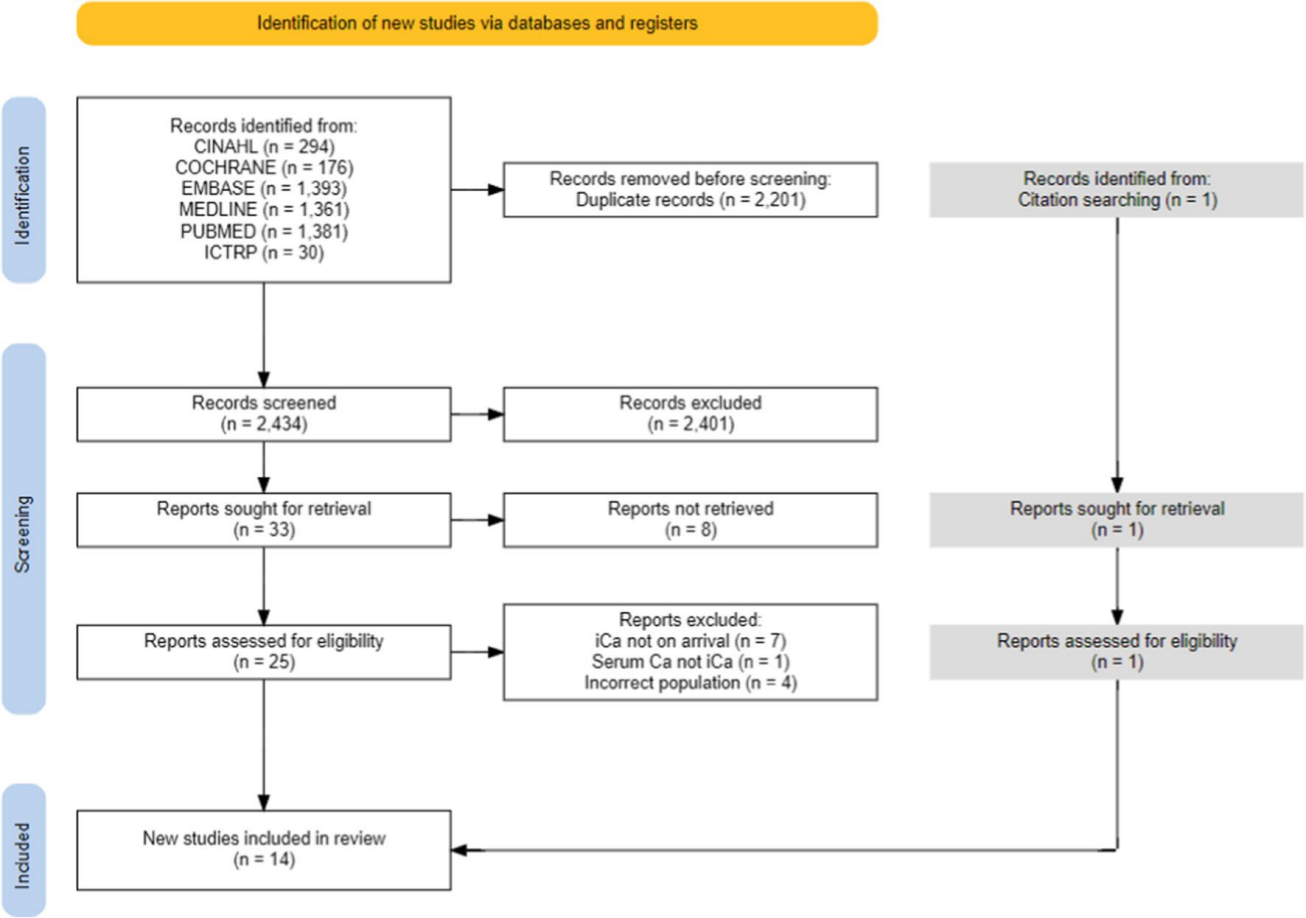
PRISMA diagram of search and inclusion processes

Seven authors were contacted [8, 11, 17, 39, 44–46] to request further information regarding inclusion criteria such as iCa measurement and injury severity. Four authors replied, resulting in the inclusion of 3 studies [8, 11, 39] and 1 study being excluded [46]. One study of hypocalcaemia in a military setting [47] initially appeared to meet criteria, however, was excluded due to unclear injury severity due to a lack of ISS reporting. The search and selection process has been outlined by a PRISMA flow diagram (Fig. 1).

### Description of the included studies

Of the 14 studies included in the systematic review, 2 of these studies consisted of a transfusion group only [38, 42], 7 consisted of a non-transfusion group only [8–11, 14, 16, 43], and 5 were comparative studies with a transfusion and non-transfusion group [15, 19, 39–41], 3 of which presented sufficient data to permit statistical analysis [15, 39, 40]. General study characteristics and risk of bias assessment are presented in Table 1. Patient characteristics such as injury severity, mechanism of injury, as well as laboratory parameters are presented in Table 2.

**Table 1:** Summary of studies reporting iCa in severe trauma patients who did and did not receive prehospital blood products

Product or no product	Author, year published	Country	Study period	Study design	Type of blood products	Definition of hypocalcaemia	Patients with iCa (*n*)	Risk of bias rating*
Product	Bodnar, 2022	Australia	Jan 2012 to Dec 2016	Retrospective	PRBC	iCa < 1.15 mmol/L	148	3 / 0 / 2
	Lyon, 2017	UK	Feb 2013 to Dec 2014	Retrospective cohort	PRBC	Not specified	109	3 / 0 / 2
No product	Vettorello, 2023	Italy	Jan 2015 to Dec 2021	Retrospective cohort	N/A	iCa < 1.11 mmol/L	798	3 / 1 / 2
	Matthay, 2020	USA	Jan 2010 to Dec 2018	Prospective observational	N/A	Not specified	88	3 / 0 / 2
	Vasudeva, 2020	Australia	Jul 2014 to Jun 2018	Retrospective cohort	N/A	iCa < 1.11 mmol/L	226	2 / 1 / 3
	Magnotti, 2011	USA	Jan 2008 to Sep 2008	Prospective observational	N/A	iCa < 1.00 mmol/L	591	3 / 1 / 3
	Choi, 2008	Korea	Jan 2005 to Dec 2005	Prospective + retrospective	N/A	iCa < 1.15 mmol/L	255	4 / 2 / 3
	Cherry, 2006	USA	Jan 2000 to Dec 2002	Retrospective	N/A	iCa < 1.00 mmol/L	396	3 / 2 / 2
	Vivien, 2005	France	Jan 2002 to Dec 2002	Prospective cohort	N/A	iCa < 1.15 mmol/L	212	3 / 2 / 3
Product vs no product	Crombie, 2022	UK	Nov 2016 to Jan 2021	Randomised controlled trial	PRBC, LyoPlas	Not specified	308	Some concerns (ROB2)
	Chanthima, 2021	USA	Oct 2016 to Sep 2018	Retrospective	PRBC, FFP	iCa < 1.18 mmol/L	93	2 / 2 / 2
	Moore, 2020	USA	Apr 2014 to Oct 2017	Post hoc analysis of PAMPER & COMBAT RCT’s	FFP, PRBC	iCa < 1.00 mmol/L	160	3 / 1 / 2
	Kyle, 2018	Afghanistan (UK military)	Jan 2010 to Dec 2014	Retrospective cohort	PRBC, FFP	iCa < 1.12 mmol/L	264	2 / 0 / 2
	Webster, 2016	UK	Jan 2013 to Dec 2014	Retrospective cohort	Assorted*	iCa < 1.10 mmol/L	63	2 / 0 / 2

*Risk of bias reported as Newcastle–Ottawa Scale (NOS); *S*, selection; *C*, comparison; and *O*, outcome unless otherwise specified. The NOS is scored from 0 to 9; scores > / = 7 were deemed to be of “good” quality, 2–6 “fair” quality, and < 2 “poor” quality

**Table 2:** Summary of patient characteristics and laboratory parameters in studies reporting iCa in severe trauma patients who did and did not receive prehospital blood products

Product or no product	Author, year published	Total cohort (*n*)	Patients with iCa (*n*)	ISS	Shock index	Blunt injury (%)	Penetrating injury (%)	iCa (mmol/L)	Lactate (mmol/L)	Mortality (%)
Product	Bodnar, 2022	164	148	33.5 (22–41)	−	78.0	22.0	1.12 (1.07–1.17)	5 (3.2–7.5)	30.49
	Lyon, 2017	147	109	32.9 (13.41)	−	87.07	9.52	1.1 (Mean)	5.27 (4.08)	64.29
No product	Vettorello, 2023	798	798	26 (21–36)	0.8 (0.4)	100	0	1.15* (Mean)	2.87* (Mean)	4.34
	Matthay, 2020	538	88	15 (Mean)	0.77 (Median)	57.95	42.05	1.08 (0.08)	4.6 (Mean)	14
	Vasudeva, 2020	226	226	32 (20.5–38.5)*	1.2** (Mean)	81.4*	14.16	iCa > 1.1 (n = 113), iCa < / = 1.1 (*n* = 113)	3.45* (Median)	20.35
	Magnotti, 2011	591	591	21 (Mean)	−	78	22	iCa > / = 1 (*n* = 259), iCa < 1 (*n* = 332)	3.9 (Mean)	13
	Choi, 2008	255	255	18.5 (9.2)	0.8** (Mean)	95.29	4.71	0.96* (Mean)	−	15.29
	Cherry, 2006	396	396	22.7 (13–34)*	−	−	−	iCa > 1 (*n* = 305), iCa < / = 1 (*n* = 91)	−	18.18
	Vivien, 2005	212	212	34 (20)	0.8** (Mean)	97.17	2.83	1.12* (Mean)	3.30* (Mean)	20.75
Product vs no product	Crombie, 2022	432	152 (Product)	36 (25–50)	1.6	78	23	1.21 (0.42)	7.04 (4.50)	43.35
			156 (No product)		1.5	80	22	1.24 (0.37)	6.93 (4.58)	45.41
	Chanthima, 2021	346	50 (Product)	32 (16)	1.1 (0.51)	−	−	1	5.8 (4.1)	26.59
			43 (No product)			−	−	1.02		
	Moore, 2020	160	76 (Product)	22 (17–34)	1.60 (1.38–1.85)	65.79	−	0.99 (0.94–1.06)	−	11.84
			84 (No product)		1.53 (1.37–1.87)	76.19	−	1.03 (0.97–1.09)	−	17.86
	Kyle, 2018	297	237 (Product)	26 (4–75)	−	−	−	1.03	−	−
			27 (No product)		−	−	−	1.2 (0.8–1.4)	−	−
	Webster, 2016	63	8 (Product)	24.6 (5.3–50)*	−	75	25	1.07	−	12.5
			55 (No product)		−	81.82	18.18	1.12	−	18.18

Outcomes reported for the total cohort which may differ in number from the cohort of patients with iCa reported. *Weighted averages calculated as required; shock index was reported in the papers or calculated by the authors of this paper based on reported arrival to ED heart rate and blood pressure. These calculations are indicated by “**”

### Meta-analysis results

**Fig. 2:**
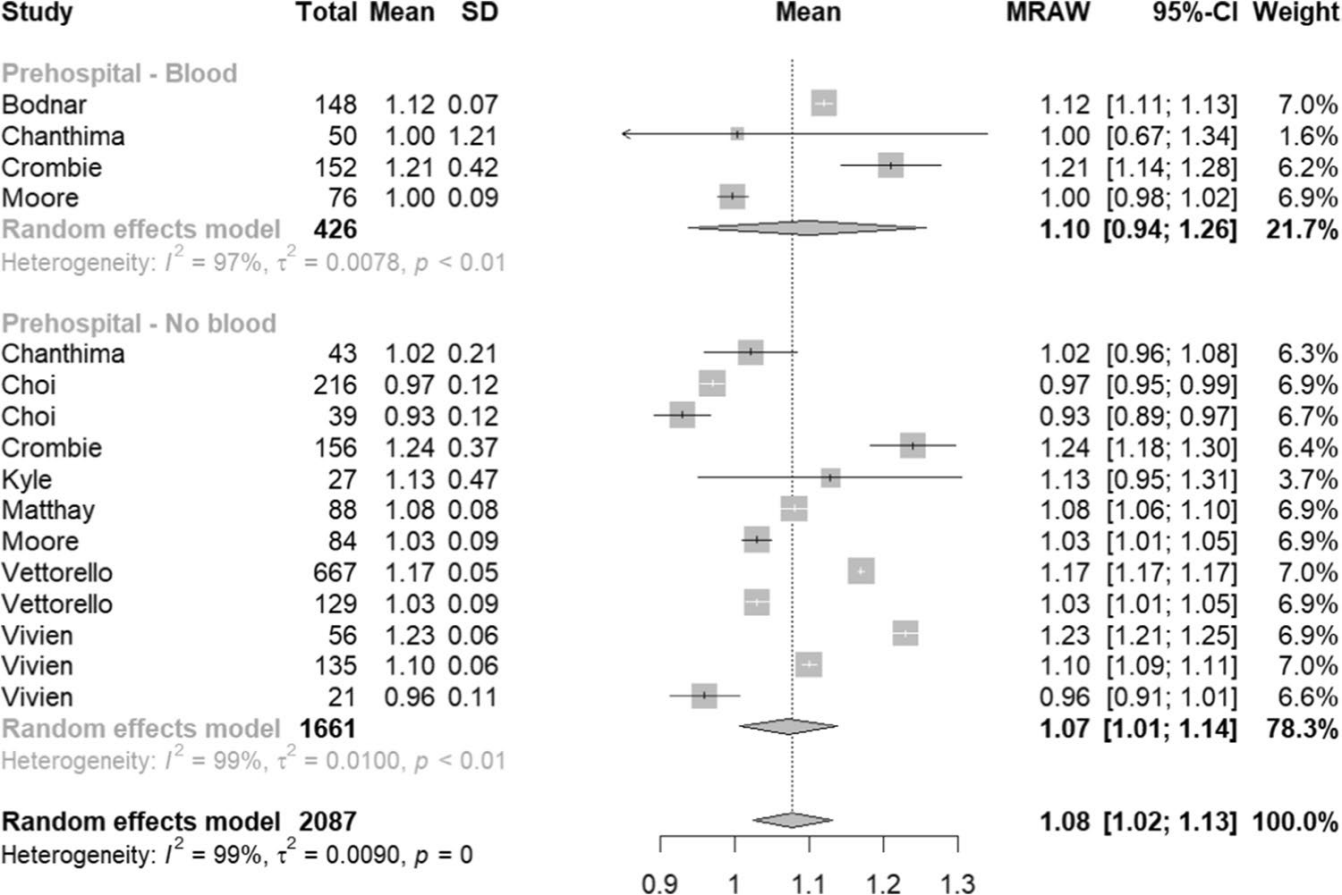
Forest plot of iCa concentration (mmol/L) on arrival to ED, grouped by whether prehospital blood was administered or not. Multiple exclusive subgroups may be present in the same study

Nine studies reported iCa on arrival to ED (Fig. 2), with a mean of 1.08 mmol/L (95% CI 1.02–1.13; *I*^2^ = 99%; 2087 patients). Subgroup analysis of patients who did not receive prehospital blood transfusion had a mean iCa of 1.07 mmol/L (95% CI 1.01–1.14; *I*^2^ = 99%, 1661 patients).

**Fig. 3:**
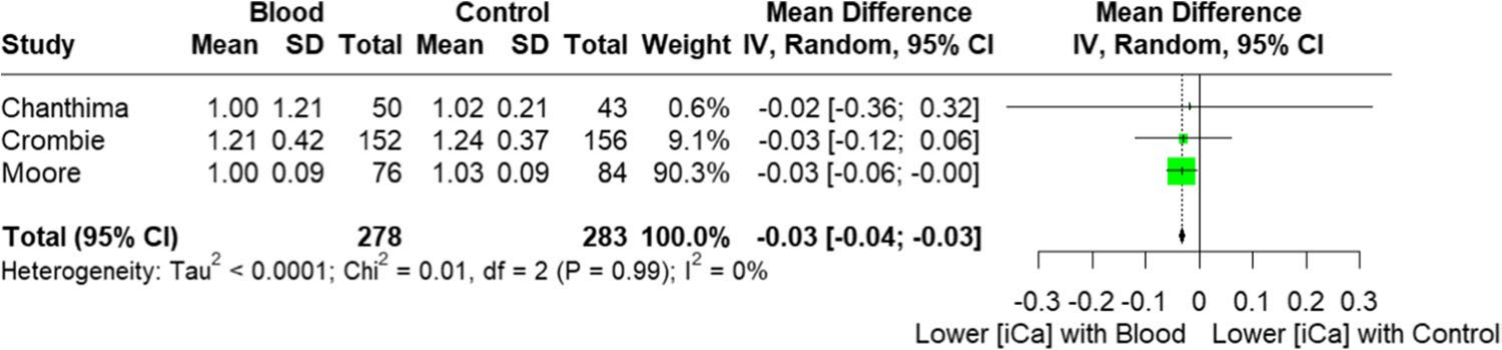
Forest plot of iCa (mmol/L) concentration on arrival to ED, comparing those who received prehospital blood transfusion versus those who did not. SD, standard difference

Patients who received prehospital blood transfusion in 3 comparative studies (Fig. 3) had a slightly lower iCa on arrival compared to those who did not receive transfusion (mean difference − 0.03 mmoL/L, 95% CI − 0.04 to − 0.03, *I*^2^ = 0%, *p* = 0.001, 561 patients). This is consistent with the result of the Bayesian meta-analysis using vague priors, with posterior probability of the MD of − 0.03 (95% CI, − 0.23 to 0.18). The posterior probability that prehospital transfusion reduced iCa on arrival to ED was 76.8%.

Eleven studies reported ISS [9–11, 15, 16, 19, 38–42] with an aggregated mean of 27 (95% CI 26.5–27.5; *I*^2^ = 98%; 2698 patients). There was significant heterogeneity, variation, and overall, not enough granularity to compare ISS for transfused vs non-transfused groups. We were not able to regress ISS against iCa outcomes either without patient-level raw data.

## Discussion

To our knowledge, this is the first systematic review and meta-analysis that compared the arrival iCa of trauma patients who received prehospital blood products with those who did not. Overall, the results highlight that hypocalcaemia is a common metabolic disturbance in the trauma patient, outside of blood transfusion. Quantitative analysis of the available comparative studies revealed a statistically significant difference (mean difference 0.03 mmol/L) (Fig. 3) between the arrival iCa of patients who did and did not receive prehospital blood products. Patients in both the blood transfusion and non-transfusion groups were hypocalcaemic upon arrival at hospital by nearly all published definitions; suggesting that whilst blood products do play a role in the pathogenesis of hypocalcaemia in this population, it is not the sole cause. This is in keeping with a recent systematic review that looked at shocked trauma patients who did not receive prehospital blood products [27]. These findings suggest that we have possibly overemphasised the concept of citrate-induced hypocalcaemia alone, especially in the context of the prehospital setting where transfusion volume is limited and hence citrate load is also limited prior to hospital arrival. The literature base has likely overlooked the concept of shock-induced hypocalcaemia, irrespective of transfusion.

Whilst the exact mechanism of hypocalcaemia in trauma is yet to be elucidated, various sources suggest potential mechanisms other than citrate chelation, such as dilution [43, 48, 49], colloid binding [50], lactate binding [21], acidosis [9, 51], ischaemic reperfusion [52–55], and impaired parathyroid hormone secretion or action [56–59]. In fact, proinflammatory cytokines such as IL-6 have been found to suppress parathyroid hormone at clinically relevant concentrations [16, 56]. Notably, IL-6 levels have been found to be significantly increased in relation to the severity of trauma [60–66]. This is of particular interest as inflammatory cytokines in the form of DAMPs (damage-associated molecular proteins) have been suggested as a potential target for immunomodulatory therapy to reduce mortality and morbidity in acute trauma and haemorrhagic shock [67, 68].

Administration of calcium in severe trauma is viewed by some as a double-edged sword. Phosphatidylserine (PtdSER), a negatively charged phospholipid plays an important role in coagulation. It is transported to the outer leaflet of the plasma membrane in platelets where it acts as a base for positively charged calcium ions to form a bridge between PtdSER and the negatively charged dicarboxyl glutamic acid residues in the vitamin K-dependent coagulation factors, thus enhancing the activation of prothrombin to thrombin [69–71]. However, calcium ions also play a role in cellular apoptosis following injury. In response to calcium-dependent stimuli, PtdSER is known to have an important role in the regulation of apoptosis. Calcium triggers the exposure of PtdSER on the outer cell membrane, which then acts as an “eat me” signal and causes apoptosis of cells [72].

In a recently published observational paper [73], the authors concluded that trauma-induced disturbances in ionized calcium levels correlate parabolically with coagulopathy, transfusion, and mortality. They concluded that “iCa2 + levels change dynamically and are more a reflection of severity of injury and accompanying physiological derangements, rather than an individual parameter that needs to be corrected as such” [73]. It is possible that hypocalcaemia could be a normal physiologic stress response to support coagulation or to limit apoptosis following injury and flooding the system with too much calcium could make things worse.

Another important finding from this review is a lack of consistency in the existing literature concerning the definition of hypocalcaemia in trauma. Various sources define hypocalcaemia from < 1 mmol/L [14–16] to < 1.2 mmol/L [47, 74] and anything in between (Table 1). Many multijurisdictional hospital MTPs use iCa targets of > 1.1 mmol/L. Consensus is needed to aid future investigations. Based on the findings of this SRMA, we recommend a standardised definition of hypocalcaemia to aid further collaborative research. Given most MTPs target iCa > 1.1 mmol/L, it would seem logical to use that as the definition of hypocalcaemia in trauma.

We also recommend that trauma centres routinely collect and record arrival iCa levels in their trauma registries to allow future collaborative research to progress. In many trauma systems/registries, this is not a routinely collected variable. Lastly, as it is clear that hypocalcaemia is an important factor in the trauma patient, we suggest that randomised controlled trials comparing the administration of calcium, trends of calcium levels, and its effect on mortality, morbidity, and transfusion demands in severe trauma are needed. Options could include (1) a prehospital RCT investigating single-dose IV calcium versus placebo in shocked trauma patients and (2) an RCT comparing two different calcium doses, in particular where up front/empiric transfusion is performed.

### Limitations

There are several limitations which make it difficult to make more robust conclusive statements, and which highlight the need for further high-quality prospective studies. As expected, there was a paucity of high-quality prehospital transfusion-related literature sources. Many were composed of small sample sizes and had a moderate-to-high risk of bias. This systematic review highlighted 5 comparative studies; however, only 3 had the required data for meta-analysis (Fig. 3). Whilst our analysis did highlight a statistically significant difference in arrival iCa between the transfusion and non-transfusion groups, the results were heavily weighted (90.3%) towards a single study [15]; a post hoc analysis of 2 prehospital plasma randomised controlled trials [75, 76] which if removed, yielded a non-significant result. Their study also utilised back calculation as a method to calculate standard deviation, an accepted yet imprecise methodology. That said, the findings of prehospital transfusion and shock-induced hypocalcaemia are undoubtedly present.

There was also significant heterogeneity amongst the studies in the reporting and definition of iCa on arrival to hospital. Blood product volume, and type, and hence citrate load, varied within and between studies due to the different policies and practices of prehospital services as well as the fact that most studies were retrospective in nature. This is common in trauma research. Interestingly, one study adjusted for citrate load by comparing the arrival iCa to the amount of citrate transfused and found no association [39]. There was also a lack of information on the prehospital phase in the studies, notably timings which intuitively should be reported from point of injury with respect to iCa levels over time.

## Conclusion

Prehospital blood transfusion and injury severity are associated with hypocalcaemia on arrival to hospital, the hypocalcaemia effect more pronounced in the former group. There is a need for an internationally accepted definition of hypocalcaemia in trauma to allow further research and benchmarking. There is also a need for routine collection of iCa levels on hospital arrival in trauma registries and correlation with other variables from point of injury such as prehospital vital signs, injury severity, prehospital times, time to haemostatic resuscitation, and haemorrhage control. This systematic review and meta-analysis provide reasonable argument and equipoise for future RCTs in calcium administration in severe trauma patients.

## References

[1] Marks AR. Calcium and the heart: a question of life and death. J Clin Investig. 2003;111:597–600. 10.1172/JCI18067. 12618512 10.1172/JCI18067 PMC151912

[2] Morgan JP, Perreault CL, Morgan KG. The cellular basis of contraction and relaxation in cardiac and vascular smooth muscle. Am Heart J. 1991;121:961–8. 10.1016/0002-8703(91)90227-9. 1848034 10.1016/0002-8703(91)90227-9

[3] Drop LJ, Scheidegger D. Plasma ionized calcium concentration. J Thorac Cardiovasc Surg. 1980;79:425–31. 10.1016/S0022-5223(19)37951-6. 7354637

[4] Diercks DB, Shumaik GM, Harrigan RA, Brady WJ, Chan TC. Electrocardiographic manifestations: electrolyte abnormalities. J Emerg Med. 2004;27:153–60. 10.1016/j.jemermed.2004.04.006. 15261358 10.1016/j.jemermed.2004.04.006

[5] Drop LJ, Geffin GA, O’Keefe DD, Newell JB, Jacobs ML, Fowler BN, et al. Relation between ionized calcium concentration and ventricular pump performance in the dog under hemodynamically controlled conditions. Am J Cardiol. 1981;47:1041–51. 10.1016/0002-9149(81)90210-1. 7223649 10.1016/0002-9149(81)90210-1

[6] Lang RM. Left ventricular contractility varies directly with blood ionized calcium. Ann Intern Med. 1988;108:524. 10.7326/0003-4819-108-4-524. 3248127 10.7326/0003-4819-108-4-524

[7] Lier H, Krep H, Schroeder S, Stuber F. Preconditions of hemostasis in trauma: a review. The influence of acidosis, hypocalcemia, anemia, and hypothermia on functional hemostasis in trauma. J Trauma Inj Infect Crit Care. 2008;65:951–60. 10.1097/TA.0b013e318187e15b. 10.1097/TA.0b013e318187e15b 18849817

[8] Matthay ZA, Fields AT, Nunez-Garcia B, Patel MH, Cohen MJ, Callcut RA, et al. Dynamic effects of calcium on in vivo and ex vivo platelet behavior after trauma. J Trauma Acute Care Surg. 2020;89:871–9. 10.1097/TA.0000000000002820. 32852184 10.1097/TA.0000000000002820 PMC7830742

[9] Vivien B, Langeron O, Morell E, Devilliers C, Carli PA, Coriat P, et al. Early hypocalcemia in severe trauma*. Crit Care Med. 2005;33:1946–52. 10.1097/01.CCM.0000171840.01892.36. 16148464 10.1097/01.ccm.0000171840.01892.36

[10] Vasudeva M, Mathew JK, Fitzgerald MC, Cheung Z, Mitra B. Hypocalcaemia and traumatic coagulopathy: an observational analysis. Vox Sang. 2020;115:189–95. 10.1111/vox.12875. 31845341 10.1111/vox.12875

[11] Choi YC, Hwang SY. The value of initial ionized calcium as a predictor of mortality and triage tool in adult trauma patients. J Korean Med Sci. 2008;23:700. 10.3346/jkms.2008.23.4.700. 18756060 10.3346/jkms.2008.23.4.700 PMC2526411

[12] Hastbacka J, Pettila V. Prevalence and predictive value of ionized hypocalcemia among critically ill patients. Acta Anaesthesiol Scand. 2003;47:1264–9. 10.1046/j.1399-6576.2003.00236.x. 14616325 10.1046/j.1399-6576.2003.00236.x

[13] Byerly S, Inaba K, Biswas S, Wang E, Wong MD, Shulman I, et al. Transfusion-related hypocalcemia after trauma. World J Surg. 2020;44:3743–50. 10.1007/s00268-020-05712-x. 32734451 10.1007/s00268-020-05712-x PMC7391918

[14] Magnotti LJ, Bradburn EH, Webb DL, Berry SD, Fischer PE, Zarzaur BL, et al. Admission ionized calcium levels predict the need for multiple transfusions: a prospective study of 591 critically ill trauma patients. J Trauma Inj Infect Crit Care. 2011;70:391–7. 10.1097/TA.0b013e31820b5d98. 10.1097/TA.0b013e31820b5d98 21307739

[15] Moore HB, Tessmer MT, Moore EE, Sperry JL, Cohen MJ, Chapman MP, et al. Forgot calcium? Admission ionized-calcium in two civilian randomized controlled trials of prehospital plasma for traumatic hemorrhagic shock. J Trauma Acute Care Surg. 2020;88:588–96. 10.1097/TA.0000000000002614. 32317575 10.1097/TA.0000000000002614 PMC7802822

[16] Cherry RA, Bradburn E, Carney DE, Shaffer ML, Gabbay RA, Cooney RN. Do early ionized calcium levels really matter in trauma patients? J Trauma Inj Infect Crit Care. 2006;61:774–9. 10.1097/01.ta.0000239516.49799.63. 10.1097/01.ta.0000239516.49799.63 17033540

[17] MacKay EJ, Stubna MD, Holena DN, Reilly PM, Seamon MJ, Smith BP, et al. Abnormal calcium levels during trauma resuscitation are associated with increased mortality, increased blood product use, and greater hospital resource consumption. Anesth Analg. 2017;125:895–901. 10.1213/ANE.0000000000002312. 28704250 10.1213/ANE.0000000000002312 PMC5918410

[18] Kronstedt S, Roberts N, Ditzel R, Elder J, Steen A, Thompson K, et al. Hypocalcemia as a predictor of mortality and transfusion. A scoping review of hypocalcemia in trauma and hemostatic resuscitation. Transfusion (Paris) 2022;62. 10.1111/trf.16965. 10.1111/trf.16965 PMC9545337 35748676

[19] Webster S, Todd S, Redhead J, Wright C. Ionised calcium levels in major trauma patients who received blood in the Emergency Department. Emerg Med J. 2016;33:569–72. 10.1136/emermed-2015-205096. 26848163 10.1136/emermed-2015-205096

[20] Ditzel RM, Anderson JL, Eisenhart WJ, Rankin CJ, DeFeo DR, Oak S, et al. A review of transfusion- and trauma-induced hypocalcemia: is it time to change the lethal triad to the lethal diamond? J Trauma Acute Care Surg. 2020;88:434–9. 10.1097/TA.0000000000002570. 31876689 10.1097/TA.0000000000002570

[21] Wray JP, Bridwell RE, Schauer SG, Shackelford SA, Bebarta VS, Wright FL, et al. The diamond of death: hypocalcemia in trauma and resuscitation. Am J Emerg Med. 2021;41:104–9. 10.1016/j.ajem.2020.12.065. 33421674 10.1016/j.ajem.2020.12.065

[22] Killen DA, Gower RE, Grogan EL, Collins HA. Course of plasma ionized calcium following rapid infusion of acid citrate dextrose solution. Surg Forum. 1969;20:95–6. 5383159

[23] Hinkle JE, Cooperman LEEH. Serum ionized calcium changes following citrated blood transfusion in anaesthetized man. Br J Anaesth. 1971;43:1108–12. 10.1093/bja/43.12.1108. 5156293 10.1093/bja/43.12.1108

[24] Zivin JR, Gooley T, Zager RA, Ryan MJ. Hypocalcemia: a pervasive metabolic abnormality in the critically ill. Am J Kidney Dis. 2001;37:689–98. 10.1016/S0272-6386(01)80116-5. 11273867 10.1016/s0272-6386(01)80116-5

[25] Dzik WH, Kirkley SA. Citrate toxicity during massive blood transfusion. Transfus Med Rev. 1988;2:76–94. 10.1016/S0887-7963(88)70035-8. 2980082 10.1016/s0887-7963(88)70035-8

[26] Li K, Xu Y. Citrate metabolism in blood transfusions and its relationship due to metabolic alkalosis and respiratory acidosis. Int J Clin Exp Med. 2015;8:6578–84. 26131288 PMC4483798

[27] Vasudeva M, Mathew JK, Groombridge C, Tee JW, Johnny CS, Maini A, et al. Hypocalcemia in trauma patients: a systematic review. J Trauma Acute Care Surg. 2021;90:396–402. 10.1097/TA.0000000000003027. 33196630 10.1097/TA.0000000000003027 PMC7850586

[28] Shand S, Curtis K, Dinh M, Burns B. Prehospital blood transfusion in New South Wales, Australia: a retrospective cohort study. Prehosp Emerg Care. 2021;25:404–11. 10.1080/10903127.2020.1769781. 32412359 10.1080/10903127.2020.1769781

[29] Brito AMP, Schreiber M. Prehospital resuscitation. Trauma Surg Acute Care Open. 2021;6:e000729. 10.1136/tsaco-2021-000729. 34041365 10.1136/tsaco-2021-000729 PMC8112406

[30] Botteri M, Celi S, Perone G, Prati E, Bera P, Villa GF, et al. Effectiveness of massive transfusion protocol activation in pre-hospital setting for major trauma. Injury. 2022;53:1581–6. 10.1016/j.injury.2021.12.047. 35000744 10.1016/j.injury.2021.12.047

[31] Moher D, Liberati A, Tetzlaff J, Altman DG. Preferred reporting items for systematic reviews and meta-analyses: the PRISMA statement. Int J Surg. 2010;8:336–41. 10.1016/j.ijsu.2010.02.007. 21603045 PMC3090117

[32] Ouzzani M, Hammady H, Fedorowicz Z, Elmagarmid A. Rayyan—a web and mobile app for systematic reviews. Syst Rev. 2016;5:210. 10.1186/s13643-016-0384-4. 27919275 10.1186/s13643-016-0384-4 PMC5139140

[33] Sterne JAC, Savović J, Page MJ, Elbers RG, Blencowe NS, Boutron I, et al. RoB 2: a revised tool for assessing risk of bias in randomised trials. BMJ. 2019;l4898. 10.1136/bmj.l4898. 10.1136/bmj.l4898 31462531

[34] Wells G, Shea B, O’Connell D, Peterson J, Welch V, Losos M, et al. The Newcastle-Ottawa Scale (NOS) for assessing the quality of nonrandomised studies in meta-analyses. 2021. https://www.ohri.ca/programs/clinical_epidemiology/oxford.asp. Accessed 23 Jul 2023.

[35] Luo D, Wan X, Liu J, Tong T. Optimally estimating the sample mean from the sample size, median, mid-range, and/or mid-quartile range. Stat Methods Med Res. 2018;27:1785–805. 10.1177/0962280216669183. 27683581 10.1177/0962280216669183

[36] Wan X, Wang W, Liu J, Tong T. Estimating the sample mean and standard deviation from the sample size, median, range and/or interquartile range. BMC Med Res Methodol. 2014;14:135. 10.1186/1471-2288-14-135. 25524443 10.1186/1471-2288-14-135 PMC4383202

[37] IntHout J, Ioannidis JP, Borm GF. The Hartung-Knapp-Sidik-Jonkman method for random effects meta-analysis is straightforward and considerably outperforms the standard DerSimonian-Laird method. BMC Med Res Methodol. 2014;14:25. 10.1186/1471-2288-14-25. 24548571 10.1186/1471-2288-14-25 PMC4015721

[38] Bodnar D, Stevens Z, Williams S, Handy M, Rashford S, Brown NJ. Hypofibrinogenaemia and hypocalcaemia in adult trauma patients receiving pre-hospital packed red blood cell transfusions: potential for supplementary pre-hospital therapeutic interventions. Emerg Med Australas. 2022;34:333–40. 10.1111/1742-6723.13887. 34706397 10.1111/1742-6723.13887

[39] Chanthima P, Yuwapattanawong K, Thamjamrassri T, Nathwani R, Stansbury LG, Vavilala MS, et al. Association between ionized calcium concentrations during hemostatic transfusion and calcium treatment with mortality in major trauma. Anesth Analg. 2021. 10.1213/ANE.0000000000005431. 33646983 10.1213/ANE.0000000000005431

[40] Crombie N, Doughty HA, Bishop JRB, Desai A, Dixon EF, Hancox JM, et al. Resuscitation with blood products in patients with trauma-related haemorrhagic shock receiving prehospital care (RePHILL): a multicentre, open-label, randomised, controlled, phase 3 trial. Lancet Haematol. 2022;9:e250–61. 10.1016/S2352-3026(22)00040-0. 35271808 10.1016/S2352-3026(22)00040-0 PMC8960285

[41] Kyle T, Greaves I, Beynon A, Whittaker V, Brewer M, Smith J. Ionised calcium levels in major trauma patients who received blood en route to a military medical treatment facility. Emerg Med J. 2018;35:176–9. 10.1136/emermed-2017-206717. 29175878 10.1136/emermed-2017-206717

[42] Lyon RM, de Sausmarez E, McWhirter E, Wareham G, Nelson M, Matthies A, et al. Pre-hospital transfusion of packed red blood cells in 147 patients from a UK helicopter emergency medical service. Scand J Trauma Resusc Emerg Med. 2017;25:12. 10.1186/s13049-017-0356-2. 28193297 10.1186/s13049-017-0356-2 PMC5307870

[43] Vettorello M, Altomare M, Spota A, Cioffi SPB, Rossmann M, Mingoli A, et al. Early hypocalcemia in severe trauma: an independent risk factor for coagulopathy and massive transfusion. J Pers Med. 2022;13:63. 10.3390/jpm13010063. 36675724 10.3390/jpm13010063 PMC9863326

[44] Giancarelli A, Birrer KL, Alban RF, Hobbs BP, Liu-DeRyke X. Hypocalcemia in trauma patients receiving massive transfusion. J Surg Res. 2016;202:182–7. 10.1016/j.jss.2015.12.036. 27083965 10.1016/j.jss.2015.12.036

[45] Hall C, Nagengast AK, Knapp C, Behrens B, Dewey EN, Goodman A, et al. Massive transfusions and severe hypocalcemia: an opportunity for monitoring and supplementation guidelines. Transfusion (Paris) 2021;61. 10.1111/trf.16496. 10.1111/trf.16496 34269436

[46] Ninokawa S, Tatum D, Toraih E, Nordham K, Ghio M, Taghavi S, et al. Elevated K/iCa ratio is an ancillary predictor for mortality in patients with severe hemorrhage: a decision tree analysis. The American Journal of Surgery. 2022;223:1187–93. 10.1016/j.amjsurg.2021.12.011. 34930584 10.1016/j.amjsurg.2021.12.011

[47] Conner JR, Benavides LC, Shackelford SA, Gurney JM, Burke EF, Remley MA, et al. Hypocalcemia in military casualties from point of injury to surgical teams in Afghanistan. Mil Med. 2021;186:300–4. 10.1093/milmed/usaa267. 33499442 10.1093/milmed/usaa267

[48] Fulgenico JP, Riou B, Devilliers C, Guesde R, Saada M, Viars P. Plasma ionized calcium in brain-dead patients. Intensive Care Med. 1995;21:832–7. 10.1007/BF01700967. 8557872 10.1007/BF01700967

[49] Sibbald WJ, Sardesai V, Wilson RF. Hypocalcemia and nephrogenous cyclic AMP production in critically ill or injured patients. J Trauma Inj Infect Crit Care. 1977;17:677–84. 10.1097/00005373-197709000-00004. 10.1097/00005373-197709000-00004 197252

[50] Massry SG. Excretion of phosphate and calcium. Arch Intern Med. 1973;131:828. 10.1001/archinte.1973.00320120068006. 4706773 10.1001/archinte.131.6.828

[51] Oberleithner H, Greger R, Lang F. The effect of respiratory and metabolic acid-base changes on ionized calcium concentration: in vivo and in vitro experiments in man and rat. Eur J Clin Invest. 1982;12:451–5. 10.1111/j.1365-2362.1982.tb02223.x. 6818032 10.1111/j.1365-2362.1982.tb02223.x

[52] Daly MJ, Elz JS, Nayler WG. Sarcolemmal enzymes and Na+ -Ca2+ exchange in hypoxic, ischemic, and reperfused rat hearts. Am J Physiol-Heart Circ Physiol. 1984;247:H237–43. 10.1152/ajpheart.1984.247.2.H237. 10.1152/ajpheart.1984.247.2.H237 6147098

[53] Odeh M. The role of reperfusion-induced injury in the pathogenesis of the crush syndrome. N Engl J Med. 1991;324:1417–22. 10.1056/NEJM199105163242007. 2020298 10.1056/NEJM199105163242007

[54] Shen AC, Jennings RB. Myocardial calcium and magnesium in acute ischemic injury. Am J Pathol. 1972;67:417–40. 5033257 PMC2032743

[55] Watts JA, Koch CD, LaNoue KF. Effects of Ca2+ antagonism on energy metabolism: Ca2+ and heart function after ischemia. Am J Physiol-Heart Circ Physiol. 1980;238:H909–16. 10.1152/ajpheart.1980.238.6.H909. 10.1152/ajpheart.1980.238.6.H909 7386650

[56] Carlstedt F, Lind L, Joachimsson PO, Rastad J, Wide L, Ljunghall S. Circulating ionized calcium and parathyroid hormone levels following coronary artery by-pass surgery. Scand J Clin Lab Invest. 1999;59:47–53. 10.1080/00365519950185995. 10206097 10.1080/00365519950185995

[57] Koch SM, Mehlhorn U, Baggstrom E, Donovan D, Allen SJ. Hypercalcitoninemia and inappropriate calciuria in the acute trauma patient. J Crit Care. 1996;11:117–21. 10.1016/S0883-9441(96)90007-6. 8891962 10.1016/s0883-9441(96)90007-6

[58] Laitinen K, Lamberg-Allardt C, Tunninen R, Karonen S-L, Tähtelä R, Ylikahri R, et al. Transient hypoparathyroidism during acute alcohol intoxication. N Engl J Med. 1991;324:721–7. 10.1056/NEJM199103143241103. 1997837 10.1056/NEJM199103143241103

[59] Zaloga GP. Hypocalcemia in critically ill patients. Crit Care Med. 1992;20:251–62. 10.1097/00003246-199202000-00014. 1737459 10.1097/00003246-199202000-00014

[60] Erkus S, Turgut A, Kalenderer O. Alterations in serum IL-6 levels in traumatized pediatric patients: a preliminary study for second hit concept. J Orthop Sci. 2022;27:440–7. 10.1016/j.jos.2020.12.023. 33549402 10.1016/j.jos.2020.12.023

[61] Gebhard F. Is interleukin 6 an early marker of injury severity following major trauma in humans? Arch Surg. 2000;135:291. 10.1001/archsurg.135.3.291. 10722030 10.1001/archsurg.135.3.291

[62] Jawa RS, Anillo S, Huntoon K, Baumann H, Kulaylat M. Analytic review: Interleukin-6 in surgery, trauma, and critical care: Part I: Basic science. J Intensive Care Med. 2011;26:3–12. 10.1177/0885066610395678. 21262749 10.1177/0885066610395678 PMC6209321

[63] Okeny PK, Ongom P, Kituuka O. Serum interleukin-6 level as an early marker of injury severity in trauma patients in an urban low-income setting: a cross-sectional study. BMC Emerg Med. 2015;15:22. 10.1186/s12873-015-0048-z. 26376825 10.1186/s12873-015-0048-z PMC4574191

[64] Ozturk H, Yagmur Y, Ozturk H. The prognostic importance of serum IL-1β, IL-6, IL-8 and TNF-α levels compared to trauma scoring systems for early mortality in children with blunt trauma. Pediatr Surg Int. 2008;24:235–9. 10.1007/s00383-007-2083-7. 18060414 10.1007/s00383-007-2083-7

[65] Stensballe J, Christiansen M, Tønnesen E, Espersen K, Lippert FK, Rasmussen LS. The early IL-6 and IL-10 response in trauma is correlated with injury severity and mortality. Acta Anaesthesiol Scand. 2009;53:515–21. 10.1111/j.1399-6576.2008.01801.x. 19317866 10.1111/j.1399-6576.2008.01801.x

[66] Strecker W, Gebhard F, Perl M, Rager J, Buttenschön K, Kinzl L, et al. Biochemical characterization of individual injury pattern and injury severity. Injury. 2003;34:879–87. 10.1016/S0020-1383(03)00022-6. 14636727 10.1016/s0020-1383(03)00022-6

[67] Namas R, Ghuma A, Hermus L, Zamora R, Okonkwo DO, Billiar TR, et al. The acute inflammatory response in trauma /hemorrhage and traumatic brain injury: current state and emerging prospects. Libyan J Med. 2008;4:97–103. 10.3402/ljm.v4i3.4824. 10.4176/090325 PMC3066737 21483522

[68] Sordi R, Nandra KK, Chiazza F, Johnson FL, Cabrera CP, Torrance HD, et al. Artesunate protects against the organ injury and dysfunction induced by severe hemorrhage and resuscitation. Ann Surg. 2017;265:408–17. 10.1097/SLA.0000000000001664. 28059970 10.1097/SLA.0000000000001664

[69] Wang J, Yu C, Zhuang J, Qi W, Jiang J, Liu X, et al. The role of phosphatidylserine on the membrane in immunity and blood coagulation. Biomark Res. 2022;10:4. 10.1186/s40364-021-00346-0. 35033201 10.1186/s40364-021-00346-0 PMC8760663

[70] Nelsestuen GL. Role of gamma-carboxyglutamic acid. An unusual protein transition required for the calcium-dependent binding of prothrombin to phospholipid. J Biol Chem. 1976;251:5648–56. 10.1016/S0021-9258(17)33107-1. 965381

[71] Bevers EM, Comfurius P, Zwaal RFA. Changes in membrane phospholipid distribution during platelet activation. Biochim Biophys Acta (BBA) – Biomembr. 1983;736:57–66. 10.1016/0005-2736(83)90169-4. 10.1016/0005-2736(83)90169-4 6418205

[72] Furuta Y, Pena-Ramos O, Li Z, Chiao L, Zhou Z. Calcium ions trigger the exposure of phosphatidylserine on the surface of necrotic cells. PLoS Genet. 2021;17:e1009066. 10.1371/journal.pgen.1009066. 33571185 10.1371/journal.pgen.1009066 PMC7904182

[73] Helsloot D, Fitzgerald M, Lefering R, Verelst S, Missant C. Trauma-induced disturbances in ionized calcium levels correlate parabolically with coagulopathy, transfusion, and mortality: a multicentre cohort analysis from the TraumaRegister DGU®. Crit Care. 2023;27:267. 10.1186/s13054-023-04541-3. 37415194 10.1186/s13054-023-04541-3 PMC10324195

[74] Escandon MA, Tapia AD, Fisher AD, Shackelford SA, Bebarta VS, Wright FL, et al. An analysis of the incidence of hypocalcemia in wartime trauma casualties. Med J (Ft Sam Houst Tex). 2022;17–21. 35373316

[75] Sperry JL, Guyette FX, Brown JB, Yazer MH, Triulzi DJ, Early-Young BJ, et al. Prehospital plasma during air medical transport in trauma patients at risk for hemorrhagic shock. N Engl J Med. 2018;379:315–26. 10.1056/NEJMoa1802345. 30044935 10.1056/NEJMoa1802345

[76] Moore HB, Moore EE, Chapman MP, McVaney K, Bryskiewicz G, Blechar R, et al. Plasma-first resuscitation to treat haemorrhagic shock during emergency ground transportation in an urban area: a randomised trial. Lancet. 2018;392:283–91. 10.1016/S0140-6736(18)31553-8. 30032977 10.1016/S0140-6736(18)31553-8 PMC6284829

