# Correction: Hypocalcaemia upon arrival (HUA) in trauma patients who did and did not receive prehospital blood products: a systematic review and meta-analysis

**DOI:** 10.1007/s00068-024-02544-5

**Published:** 2024-07-09

**Authors:** Timothy J. Rushton, David H. Tian, Aidan Baron, John R. Hess, Brian Burns

**Affiliations:** 1 School of Medicine Sydney, University of Notre Dame Australia, Sydney, NSW Australia; 2 Department of Anaesthesia and Perioperative Medicine, Westmead Hospital, Sydney, NSW Australia; 3 Department of Surgery, University of Melbourne, Melbourne, VIC Australia; 4 Faculty of Health, Science, Social Care and Education, Kingston University, London, UK; 5 Transfusion Service, Harborview Medical Center, Seattle, WA USA; 6 Department of Laboratory Medicine and Pathology, University of Washington School of Medicine, Seattle, WA USA; 7 Trauma Service, Royal North Shore Hospital, Reserve Rd, St Leonards, Sydney, NSW 2065 Australia; 8 Aeromedical Operations, NSW Ambulance, Sydney, NSW Australia; 9 Sydney Medical School, Sydney University, Sydney, NSW Australia; 10 Faculty of Medicine, Macquarie University, Sydney, NSW Australia

**Correction: European Journal of Trauma and Emergency Surgery**

10.1007/s00068-024-02454-6

The original version of this article, published on 06 February 2024, unfortunately contained a mistake.

In this article ref. 53 was incorrect and should have been ‘Odeh M. The role of reperfusion-induced injury in the pathogenesis of the crush syndrome. N Engl J Med. 1991;324:1417–22. 10.1056/NEJM199105163242007’.

The original article has been corrected.

